# Impact of vaccine effectiveness and coverage on preventing large mumps outbreaks on college campuses: Implications for vaccination strategy

**DOI:** 10.1016/j.epidem.2022.100594

**Published:** 2022-06-17

**Authors:** Michael Melgar, Bryan Yockey, Mariel Asbury Marlow

**Affiliations:** Division of Viral Diseases, National Center for Immunization and Respiratory Diseases, Centers for Disease Control and Prevention, Atlanta, GA 30333, USA

**Keywords:** Mumps, Disease outbreaks, Vaccine effectiveness, Vaccination, Compartmental model

## Abstract

Recent mumps outbreaks among highly vaccinated populations, including college students, have called into question the vaccine effectiveness (VE) of routine two-dose measles, mumps, and rubella (MMR2) immunization. We aimed to estimate the VE required for a novel vaccination strategy (e.g., MMR booster dose, novel vaccine) to prevent large mumps outbreaks on college campuses. Using mumps college outbreak data reported to the U.S. Centers for Disease Control and Prevention during 2016–2017, we estimated current MMR2 VE using the screening method and implemented a compartmental model of mumps transmission. We performed 2000 outbreak simulations, following introduction of an infectious person to a population of 10,000, over ranges of MMR2 vaccine coverage (VC) and VE (30.0–99.0%). We compared the impact of varying VC and VE on mumps and mumps orchitis case counts and determined VE thresholds that ensured < 5.0% and < 2.0% of the outbreak simulations exceeded 20 and 100 mumps cases. Median estimated MMR2 VE in reported mumps outbreaks was 60.5% and median reported MMR2 VC was 97.5%. Simulated mumps case count was more sensitive to changes in VE than in VC. The opposite was true for simulated mumps orchitis case count, though orchitis case count was small (mean <10 cases across simulations for VE near 60.5% and VC near 97.5%). At 97.5% VC, 73.1% and 78.2% VE were required for < 5.0% and < 2.0% of outbreaks, respectively, to exceed 100 mumps cases. Maintaining 97.5% VC, 82.4% and 85.9% VE were required for < 5.0% and < 2.0% of outbreaks, respectively, to exceed 20 cases. We conclude that maintaining current levels of MMR2 VC, a novel vaccination strategy aimed at reducing mumps transmission must achieve at least 73.1–85.9% VE among young adults to prevent large mumps outbreaks on college campuses.

## 1. Introduction

Mumps is a viral vaccine-preventable disease transmitted through respiratory droplets and direct contact with saliva. While mumps typically presents as inflammation of the parotid (parotitis) or other salivary glands, it can also cause complications including orchitis (inflammation of the testes), oophoritis (inflammation of the ovaries), mastitis, hearing loss, meningitis, and encephalitis. Prior to the introduction of the live-attenuated mumps vaccine in 1967, mumps was considered a universal childhood disease in the United States and a leading cause of aseptic meningitis and encephalitis (Galazka et al., 1999). Incidence of mumps and its complications fell dramatically beginning in 1977 with routine use of a single-dose measles, mumps, and rubella (MMR) vaccine for children aged 12 months, but outbreaks among vaccinated children and adolescents continued to occur, particularly during 1985–1992 (van Loon et al., 1995). Due to similar trends in measles incidence, a second MMR dose (MMR2) was recommended in 1989 for children aged 4–6 years. By the early 2000s, national reported mumps cases fell by 99% to < 300 cases annually (Hopkins et al., 2005) and by 2019, > 90% of adolescents in the United States had received at least two doses of MMR (Elam-Evans et al., 2020).

Since 2006, however, the reported number of mumps cases and outbreaks has increased, with several peak years. Large outbreaks have occurred in different congregate settings including universities and schools, among athletic teams and in their facilities, and within religious communities (Albertson et al., 2016; Cardemil et al., 2017; Centers for Disease Control and Prevention, 2010; Clemmons et al., 2019; Cortese et al., 2008; Nelson et al., 2013), with most outbreak-associated cases occurring among fully vaccinated people, particularly young adults. Mumps resurgence among highly vaccinated populations has been postulated to be caused by lower levels of vaccine-induced antibodies against the circulating wild-type genotype G virus strains compared with the live-attenuated vaccine genotype A virus strain (antigenic mismatch) (Centers for Disease Control and Prevention, 2006; Peltola et al., 2007), or from decreased immunity over time following vaccination (waning immunity) (Lewnard and Grad, 2018; Seagle et al., 2018), both of which may reduce vaccine effectiveness (VE). Since 2005, across 13 studies with varying methodology and setting, the MMR2 VE against clinical mumps disease has been estimated to be 32–95% (median 88%) (World Health Organization, 2020). Estimates < 80% have been primarily among young adults with the last MMR dose > 10 years prior (Greenland et al., 2012; Marin et al., 2008; Snijders et al., 2012; Vygen et al., 2016). Nonetheless, high vaccine coverage (VC) with even low-to moderate-effectiveness has been shown to suppress transmission of other respiratory viruses, such as influenza (Sah et al., 2018). MMR2 VC > 90% may similarly be suppressing national mumps incidence despite a reduced VE in certain populations, which may permit breakthrough outbreaks (Connell et al., 2020).

In 2017, the Advisory Committee on Immunization Practices recommended a third dose of MMR (MMR3) for use during mumps outbreaks (Marin et al., 2018); however, the MMR3 intervention was not intended to prevent outbreaks and is resource intensive (Marlow et al., 2020). To prevent mumps outbreaks in highly vaccinated populations, proposed vaccination strategies include administration of a universal MMR3 in late adolescence, prior to college enrollment (Lewnard and Grad, 2018; Plotkin, 2018), and implementation of a novel mumps vaccine genotype matched to circulating wild-type strains, either replacing the Jeryl Lynn strain in the currently licensed MMR vaccine or as a monovalent booster vaccine (Connell et al., 2020). However, the performance characteristics (e.g., incremental VE, durability of immunity) required for these vaccination strategies to successfully reduce mumps outbreak size and prevent mumps complications have not been described.

To compare the relative impact of MMR2 VE and population VC on mumps outbreak size, we developed a dynamic compartmental model of mumps transmission and vaccination informed by characteristics of college outbreaks reported to the U.S. Centers for Disease Control and Prevention (CDC). College campus outbreaks were chosen to characterize mumps transmission dynamics because of the well-defined population at risk and because administrative recordkeeping facilitated ascertainment of key epidemiologic variables. We used the same model to evaluate case counts of mumps orchitis, the most common complication of mumps virus infection after salivary gland involvement, and to determine VE thresholds required for a new vaccination strategy to prevent large mumps outbreaks.

## 2. Methods

Using the screening method (Orenstein et al., 1985), we first estimated MMR2 VE among college students from data reported to CDC from mumps outbreaks that occurred during January 2016–June 2017. During this period, through the Epidemiology and Laboratory Capacity cooperative agreement with CDC, 39 U.S. jurisdictional health departments (38 states and the District of Columbia) voluntarily reported enhanced surveillance data on mumps outbreaks in addition to routine case-based surveillance through the National Notifiable Diseases Surveillance System, which records patient-level variables including vaccination status, diagnostic testing, and clinical signs and symptoms. Additional outbreak-level variables collected included, when available, outbreak setting (e.g., college campus), population VC, and outbreak control interventions implemented. For inclusion in VE estimation, outbreaks had to fulfill certain criteria allowing for application of the screening method. First, student population-level pre-outbreak MMR2 VC must have been reported. Second, we required at least one mumps case to have occurred in an under-vaccinated person. Third, we excluded outbreaks for which control measures included MMR vaccination campaigns, which can rapidly change VC, unless mumps incidence data were available for ≥ 14 days pre-intervention. For outbreaks with ≥ 14 days of pre-intervention data available, only pre-intervention data were used in VE estimation. We applied the 2012 Council of State and Territorial Epidemiologists case definition for mumps, requiring confirmed or probable classification for inclusion as a mumps case (Centers for Disease Control and Prevention, 2012). MMR2 VE was estimated relative to under-vaccination (0–1 MMR doses) because population-level VC was reported as the proportion of the population who had received ≥ 2 MMR doses; counts of 1-dose recipients could not be stratified from counts of unvaccinated persons. Similarly, counts of persons with > 2 MMR doses could not be stratified from counts of 2-dose recipients, but the number of these students was likely small (Cardemil et al., 2017). VE estimates were not adjusted for covariates. Uncertainty around the VE point estimate was determined using the Wald interval, with truncation at VE of zero.

To evaluate representativeness of outbreaks with sufficient data to estimate MMR2 VE, we compared characteristics of included and excluded outbreaks. Evaluated characteristics included number of mumps cases, outbreak duration, population MMR2 VC, proportion of mumps case-patients who had received ≥ 2 MMR doses, and whether outbreak control measures included an MMR vaccination campaign. The Wilcoxon rank-sum test was used to compare continuous variables and Barnard’s exact test was used to compare dichotomous variables. P-values < 0.05 were considered statistically significant.

**Fig. 1.**
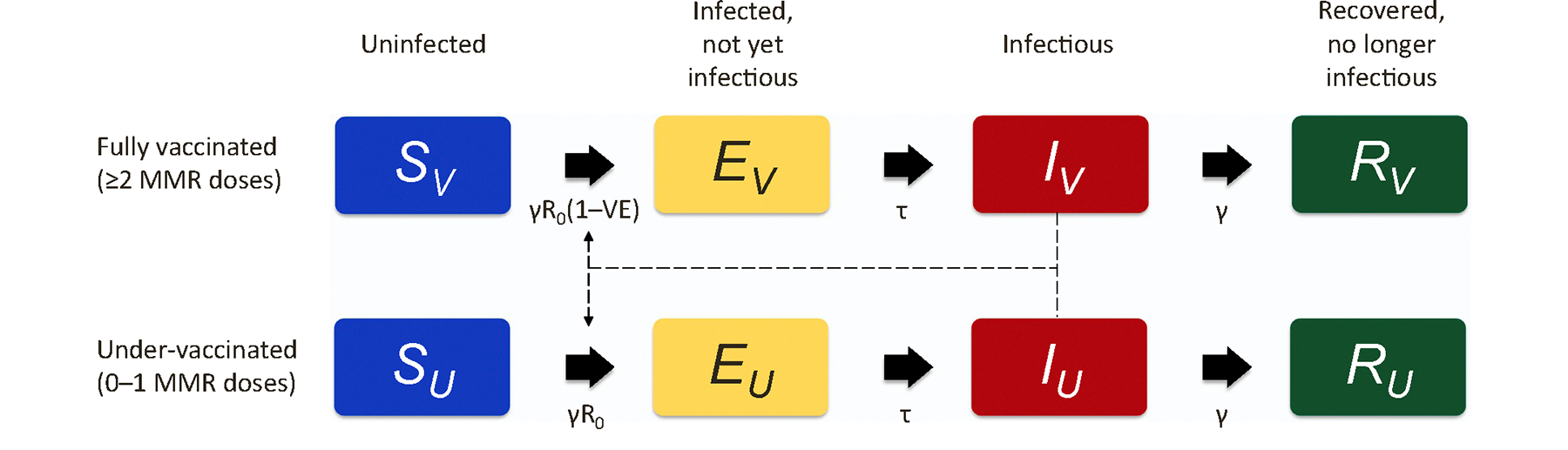
Schematic diagram of susceptible, exposed, infected, recovered (SEIR) compartmental model of mumps transmission with stratification by vaccination status. MMR indicates measles, mumps, and rubella vaccine. At a given timepoint in model simulation, S compartments represent the number of people as yet uninfected with mumps virus. E compartments represent the number of people infected with mumps virus, but who are not yet infectious. I compartments represent the number of people infectious with mumps virus. R compartments represent the number of people recovered from mumps; these people are no longer infectious and are no longer at risk of mumps infection. Compartments with a V subscript include only fully vaccinated people, while compartments with a U subscript include only under-vaccinated people. The recovery rate from infectious mumps (reciprocal of duration of infectiousness) is given by γ. The mumps virus basic reproduction number is given by R_0_. The vaccine effectiveness of full vaccination, relative to under-vaccination, against mumps is given by VE. The rate of progression from infection to infectiousness with mumps virus (reciprocal of latency time) is given by τ. Dashed lines with arrows indicate that the rates of progression from uninfected compartments S_V_ and S_U_ to infected compartments E_V_ and E_U_ are proportional to the number of infectious people (I_V_+I_U_).

We developed a susceptible, exposed, infected, recovered (SEIR) deterministic compartmental model of mumps transmission during a college outbreak (Fig. 1). Building on a published SEIR model of influenza transmission (Sah et al., 2018), we stratified compartments by vaccination status (≥2 MMR doses, 0–1 MMR doses). Compartments were not stratified by age. The daily rate of new infections was proportional to the daily number of as-yet uninfected individuals, the daily number of infectious individuals, and a basic reproduction number (R_0_) for mumps virus. Among fully vaccinated persons, the daily rate of new infections was also proportional to one minus the MMR2 VE. Ordinary differential equations governing transmission dynamics and state transitions between compartments are shown in Supplement Fig. S1.

We projected number of cases of mumps and mumps orchitis over 2000 model outbreak simulations. Each simulation was parameterized by drawing from distributions of latency (time between infection with mumps virus and onset of infectiousness, median 13.4 days, interquartile range [IQR] 12.5–14.3 days) (Henle et al., 1948) and duration of infectiousness (median 6.2 days, IQR 4.5–8.3 days) (Supplement Table S1) (Ennis and Jackson, 1968; Henle et al., 1948; Shope and Hierholzer, 1969). These estimates were consistent with previous findings that mumps virus shedding persists after the onset of parotitis in a substantial proportion of cases (Polgreen et al., 2008). For each simulation, R_0_ was calculated by first selecting one reported college outbreak, sampled with equal probability from the same set included in VE estimation. Then, a binary search algorithm was applied to calculate R_0_ required to achieve the reported outbreak size, conditional on the estimated outbreak MMR2 VE, the reported outbreak MMR2 VC, and the sampled latency and duration of infectiousness. Because R_0_ calculation required knowledge of outbreak-specific parameters including MMR2 VE, outbreaks for which VE could not be estimated were also excluded from model parameterization. Governed by the sampled latency and duration of infectiousness, and by the calculated R_0_, each simulation started with a single infectious student introduced into a population of 10,000 and lasted 62 days, equal to the median reported duration of college outbreaks without an MMR outbreak dose intervention, followed by an interruption in mumps transmission (e.g., winter or summer holidays). Each simulation was repeated for every combination of MMR2 VE and VC, each varying 30.0–99.0% in increments of 0.1%. In keeping with the assumptions required to estimate VE using the screening method, we assumed VC was fixed throughout the outbreak simulation (i.e., all vaccinations took place prior to the outbreak), so persons did not transition between under-vaccinated and fully vaccinated compartments.

For each simulation, we calculated the total number of mumps cases stratified by vaccination status, and the number of cases of mumps orchitis assuming 50% of mumps cases occurred in males. We assumed a 6.0% risk of orchitis among vaccinated post-pubertal males with mumps and 30.0% among unvaccinated males with mumps (Marlow et al., 2021). To compare the relative impact of MMR2 VC and VE, mean number of cases of mumps and mumps orchitis across simulations were calculated for each VC/VE pair, as well as the mean proportion of mumps cases occurring among fully vaccinated persons (≥2 MMR doses).

To inform VE thresholds required for a novel vaccination strategy to prevent large mumps outbreaks, we drew from the same set of 2000 simulations to calculate the proportion exceeding 20 and 100 mumps cases (large (Clemmons et al., 2019) and very large outbreaks, respectively). We then determined the novel VE required for < 5.0% and < 2.0% of simulations to exceed these outbreaks sizes. Estimates were converted from absolute novel VE (versus under-vaccination) to incremental VE (versus MMR2), using as a baseline the median estimated MMR2 VE from reported college outbreaks. Incremental VE was computed as

$$VE_{\mathrm{incremental}}=1-\frac{1\;-\;VE_{\mathrm{novel}}}{1\;-\;VE_{MMR2}}.$$

All analyses were conducted using R version 3.4.4. Ordinary differential equations were solved numerically using the LSODA routine (Hindmarsh, 1992) implemented through the ‘deSolve’ software package.

This activity was reviewed by CDC and was conducted in consistence with applicable law and CDC policy (United States Department of Health and Human Services, 2022).

## 3. Results

**Fig. 2.**
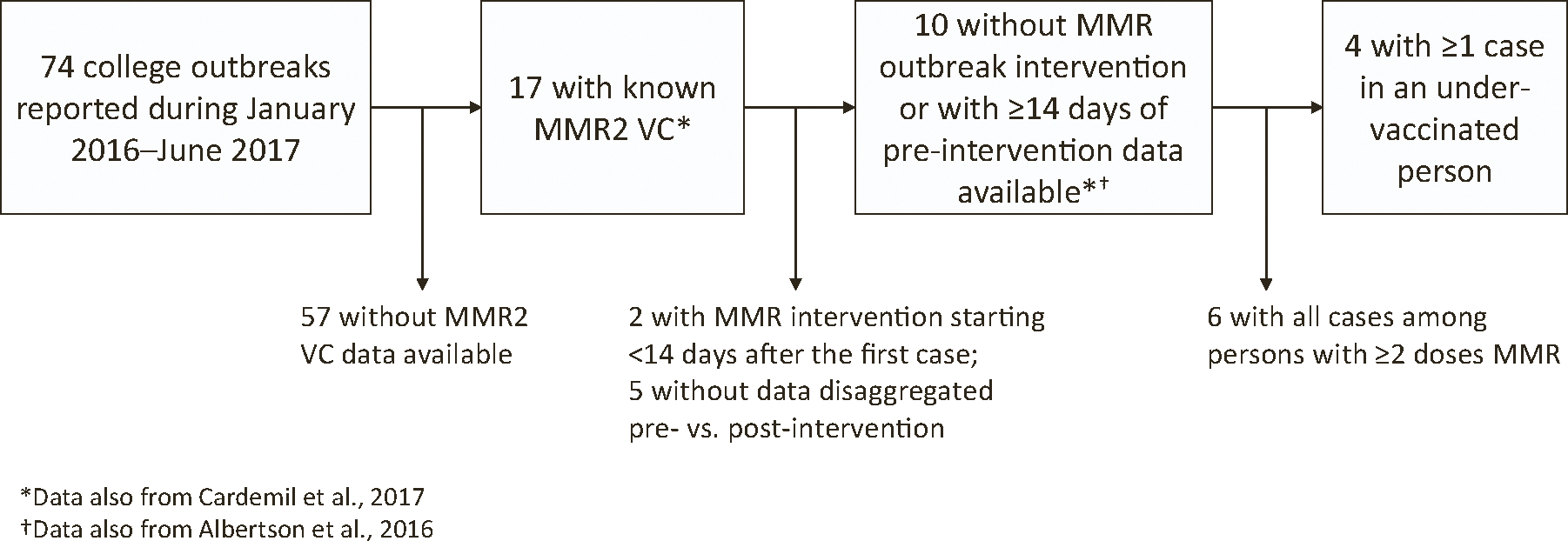
Inclusion of mumps college outbreaks in estimation of two-dose measles, mumps, and rubella vaccine effectiveness among those reported to the Centers for Disease Control and Prevention during January 2016–June 2017. VC indicates vaccine coverage. MMR indicates measles, mumps, and rubella vaccine.

During January 2016–June 2017, 74 mumps outbreaks on college campuses were reported to CDC, including 28 (37.8%) with > 20 cases and five (6.8%) with > 100 cases. Data sufficient to estimate VE using the screening method were available for only four outbreaks (Fig. 2). These four outbreaks had a greater number of reported mumps cases and longer duration than excluded outbreaks (Table 1). However, there was no statistically significant difference in population MMR2 VC, in percentage of mumps case-patients who had received ≥ 2 MMR doses, or in use of an MMR vaccination campaign for outbreak control. Across these four outbreaks, median reported MMR2 VC was 97.5% and median point estimate of MMR2 VE against clinical mumps was 60.5% (Table 2). When these outbreaks were used to parameterize 2000 outbreak simulations, median R_0_ was 3.8 (IQR: 2.6–5.5) (Supplement, Fig. S2).

**Fig. 3.**
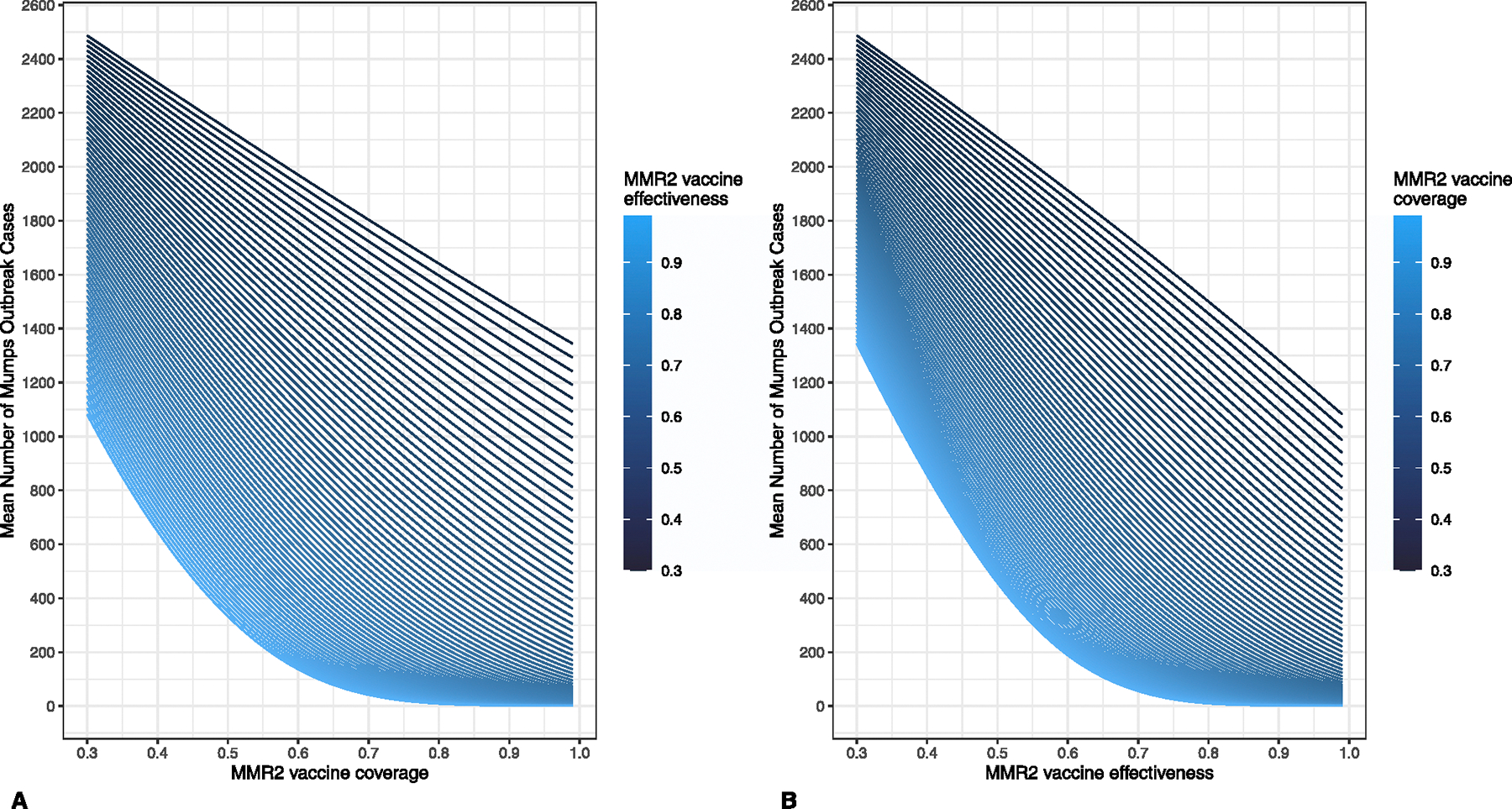
Impact of two-dose measles, mumps, rubella vaccine coverage and effectiveness against mumps on mean number of mumps cases across 2000 outbreak simulations following introduction of an infected student to a college campus of population 10,000, (A) by vaccine coverage stratified by effectiveness and (B) by vaccine effectiveness stratified by coverage. MMR2 = two doses of measles, mumps, rubella vaccine.

Across the outbreak simulations, at MMR2 VC 97.5% and VE 60.5%, mean projected number of mumps cases was 196.0 and mean projected number of mumps orchitis cases was 7.1. At VC and VE near these values, the number of mumps cases was more sensitive to changes in MMR2 VE than in VC (Fig. 3); increasing MMR2 VC from 96.0% to 99.0% with a constant VE of 60.5% reduced mean number of mumps cases by 34.8, while increasing MMR2 VE by the same amount, from 59.0% to 62.0%, with a constant VC of 97.5% resulted in a reduction of 59.4 cases (Supplement, Table S2).

Improvements in MMR2 VE also achieved greater reductions than equivalent improvements in VC in number of mumps cases occurring among fully vaccinated persons (Supplement, Fig. S3). In fact, at low VE or low VC, improvements in VC resulted in increased number of mumps cases among fully vaccinated persons, owing to increases in the total number of persons in this subgroup. In contrast, improvements in MMR2 VC and VE both resulted unequivocally in reductions in mumps cases among under-vaccinated persons, with greater reduction achieved by improvements in VC than equivalent improvements in VE (Supplement, Fig. S4). As a result, changes in MMR2 VC and VE had opposing effects on the proportion of cases occurring among fully vaccinated persons (Supplement, Fig. S5). Holding MMR2 VE constant, increases in VC resulted in a greater mean proportion of cases among vaccinated persons across simulations. Conversely, holding MMR2 VC constant, increases in VE resulted in a shift in remaining case burden away from fully vaccinated persons to under-vaccinated persons.

**Fig. 4.**
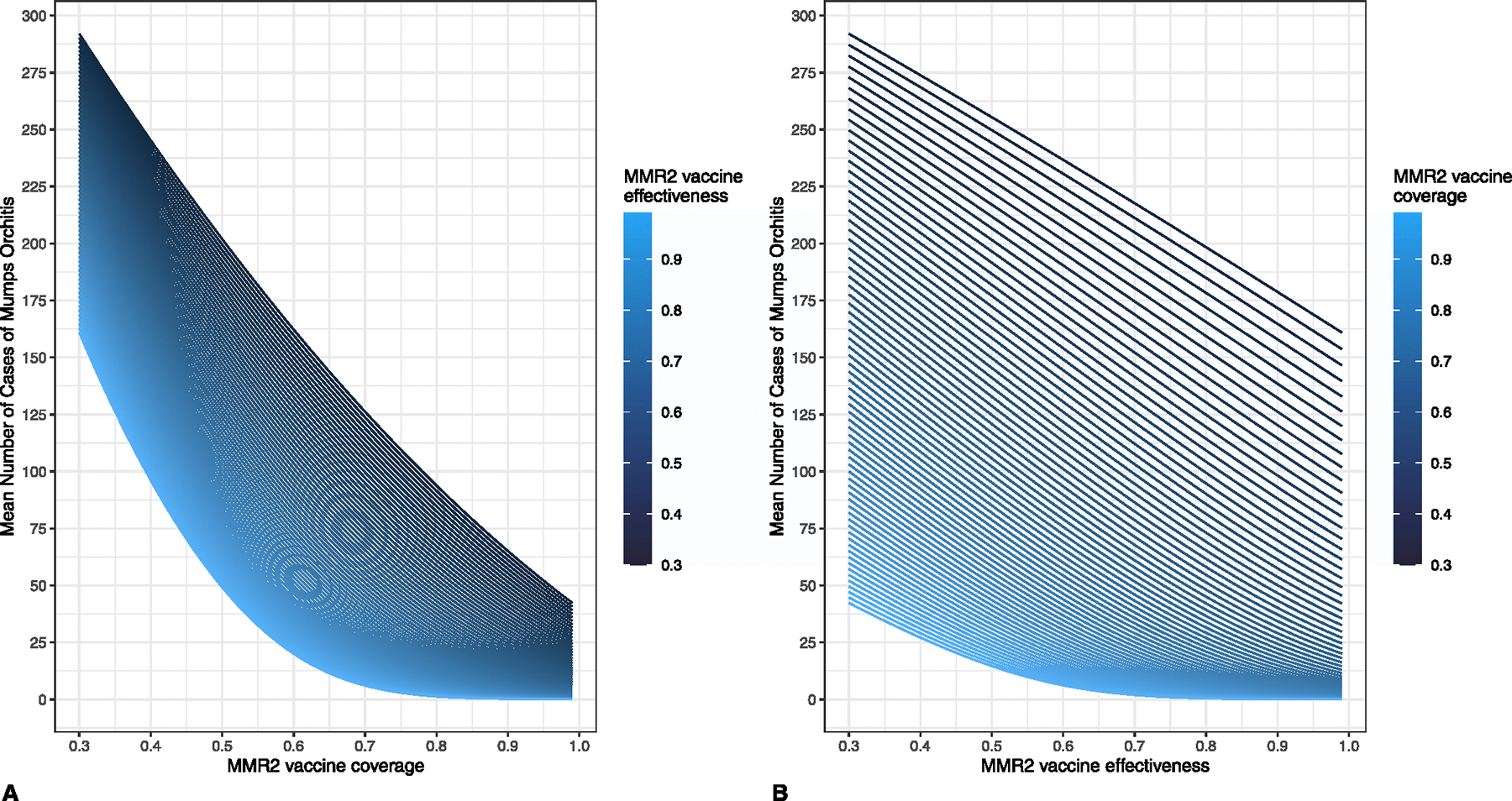
Impact of two-dose measles, mumps, rubella vaccine coverage and effectiveness against mumps on mean number of cases of mumps orchitis across 2000 outbreak simulations following introduction of an infected student to a college campus of population 10,000, (A) by vaccine coverage stratified by effectiveness and (B) by vaccine effectiveness stratified by coverage. MMR2 = two doses of measles, mumps, rubella vaccine.

Mumps orchitis case count was more sensitive to changes in MMR2 VC than in VE (Fig. 4). However, with changes near the median observed MMR2 VC and VE, absolute changes in orchitis case counts were small; increasing MMR2 VC from 96.0% to 99.0% with a constant VE of 60.5% reduced mean orchitis cases by 2.5, while increasing MMR2 VE from 59.0% to 62.0%, with a constant VC of 97.5%, reduced mean orchitis cases by 2.0 (Supplement, Table S2).

**Fig. 5.**
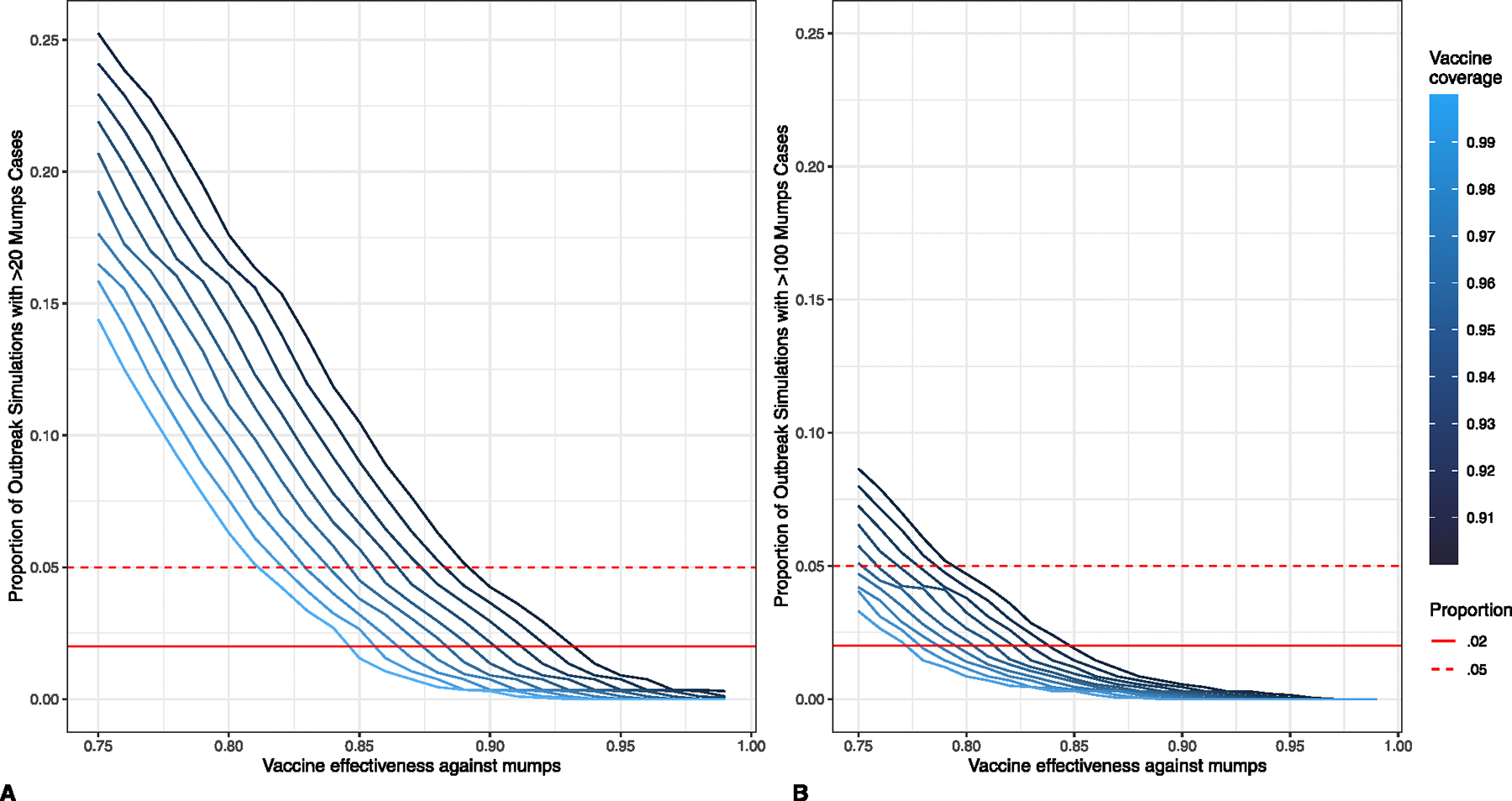
Proportion of 2000 outbreak simulations exceeding (A) 20 mumps cases and (B) 100 mumps cases on a college campus of population 10,000, by theoretical novel vaccine effectiveness against mumps, stratified by vaccine coverage. Solid red lines indicate 2% of outbreaks and dashed red lines indicate 5% of outbreaks.

Within an expected range of 90.0–99.0% for VC among college students, we used the 2000 outbreak simulations to estimate the VE required for a novel vaccination strategy to ensure that < 5.0% and < 2.0% of outbreak simulations exceeded 20 and 100 mumps cases (Fig. 5). At 97.5% VC, 73.1% VE versus under-vaccination (31.9% incremental VE versus MMR2) ensured that < 5.0% of simulations exceeded 100 cases and 78.2% VE (44.8% incremental VE) ensured that < 2.0% of simulations exceeded 100 cases (Table 3). To ensure that < 5.0% and < 2.0% of simulations exceeded 20 cases, 82.4% and 85.9% VE (55.4% and 64.3% incremental VE) was required, respectively.

## 4. Discussion

Despite sustained high MMR coverage among children and adolescents in the United States, there has been a resurgence in reported mumps cases in the last two decades, particularly among fully vaccinated young adults and in the setting of college outbreaks. The increase in cases and outbreaks suggest waning of vaccine-induced immunity or antigenic mismatch between the vaccine and circulating strains, leading to lower VE. Based on reported outbreak case counts, we estimate that the MMR2 VE among college students is substantially lower than the published estimate of 88% (World Health Organization, 2020), but is similar to previous estimates (55–79%) among adults (Greenland et al., 2012; Marin et al., 2008; Snijders et al., 2012; Vygen et al., 2016). Marked heterogeneity in outbreak-specific MMR2 VE estimates in this study (31.6–76.2%) may have resulted from varying time since last MMR dose among vaccinated students (Bianchi et al., 2020; Cardemil et al., 2017), or unstable estimates due to low proportion of under-vaccinated cases. Our results also demonstrate that MMR2 VE has a greater relative impact on mumps outbreak size than does MMR2 VC. It is likely not feasible to further increase VC in a highly vaccinated population such as college students, of whom > 95% typically have received two doses of MMR. However, while maintaining the current high VC among college students, moderate increases in VE through novel vaccination strategies may substantially reduce mumps cases and outbreaks on college campuses.

We estimate that a novel vaccination strategy aimed at preventing large (>20 cases) mumps outbreaks, whether through a universal MMR3 booster dose or a new vaccine aimed at overcoming antigenic mismatch, must increase VE to 82.4–85.9% relative to under-vaccination, representing an incremental 55.4–64.3% VE relative to MMR2. Further, the intervention must maintain this protection over at least the four years of college attendance. We estimate this improved VE would reduce large mumps outbreaks to 2–5% of those currently reported, equating to an expected 1–2 annually. A more modest VE increase to 73.1–78.2%, representing an incremental 31.9–44.8% VE, would reduce very large outbreaks with > 100 cases to 2–5%. Reducing the number of large and very large college outbreaks would reduce illnesses and complications among college students and university workers. It would also lessen the burden on universities and local and state health departments.

Following the recommendation for an MMR3 during mumps outbreaks, some experts have proposed building on this to recommend a universal MMR3 prior to college entry. In outbreak settings, the incremental MMR3 VE relative to MMR2 has been estimated to be 78.1% by 28 days post-vaccination (Cardemil et al., 2017), surpassing the thresholds we estimate would prevent most large outbreaks. However, the duration of added protection from MMR3, whether in the setting of an outbreak or universal immunization, is unknown. Although there is no established immunologic correlate of protection against mumps, serum binding and neutralizing antibody titers have been shown to re turn to near pre-MMR3 baseline by one year following MMR3 (Fie belkorn et al., 2014; Kaaijk et al., 2022; Latner et al., 2017). If the incremental VE conferred by MMR3 wanes over a similar timescale as immunogenicity, then a universal MMR3 at college entry would not maintain the VE needed to prevent large outbreaks over the course of four years of college attendance. On the other hand, the rate of antibody titer decline may plateau between one and three years post-MMR3, and MMR2 recipients with the lowest neutralizing antibody titers (presumably with the lowest degree of protective immunity) have been shown to experience the greatest increase in seropositivity after MMR3 (Kaaijk et al., 2022; Latner et al., 2017). Additional investigation is needed to evaluate MMR3 VE against mumps at timepoints beyond one month.

Evidence is mounting that antigenic mismatch also plays a role in decreased VE of the Jeryl-Lynn strain of live attenuated vaccine (genotype A) used in the MMR vaccine in the United States. From 2015–2017, nearly 99% of sequenced isolates from U.S. mumps cases belonged to genotype G (McNall et al., 2020). While vaccine-induced antibodies can neutralize genotype G strains (Rubin et al., 2008), neutralizing antibody titers in young adults have been found to be 6-fold lower against genotype G than against the vaccine strain (Rasheed et al., 2019). Adoption of a new genotype G-containing mumps vaccine has been proposed to address potential antigenic mismatch, either replacing the Jeryl-Lynn strain in the existing MMR vaccine, or as a monovalent booster. Although the clinical significance of the difference in neutralizing antibody titer has not been established, our study provides a target VE that a novel genotype G vaccine would need to achieve and maintain through young adulthood to successfully prevent large college outbreaks.

We found mumps outbreak size to be more sensitive to changes in MMR2 VE than in VC. While comparable data regarding the relative importance of VE and VC to mumps transmission have been lacking, our results stand in contrast to findings from similar compartmental models of influenza transmission on a national level (Hughes et al., 2020; Sah et al., 2018). This may in part be due to the age homogeneity in our model; age structured models of influenza transmission have demonstrated the importance of population VC in reducing hospitalizations and mortality, which primarily affect individuals older than 65 years. These reductions are in part attributed to higher VC among older adults themselves, but also to herd immunity effects, reducing transmission from younger people (Sah et al., 2018). Influenza VC tends to be lower among younger age groups in the United States, who also benefit from reduced incidence with higher VC (Hughes et al., 2020). College students in the United States, however, are highly vaccinated against measles, mumps, and rubella. Therefore, even small increases in MMR2 VE would result in greater reductions in number of mumps cases in this demographic than would comparable improvements in VC.

We found that, unlike mumps incidence, incidence of mumps orchitis was more sensitive to changes in MMR2 VC than in VE. While improvements in both MMR2 VC and VE resulted in reduced mumps incidence, improvement in MMR2 VC resulted in an increase in the proportion of mumps cases occurring in vaccinated persons, who are still protected against mumps complications such as orchitis in men. The reverse was true for improvements in MMR2 VE; the proportion of mumps cases occurring in under-vaccinated persons increased. Under-vaccinated people with mumps have increased vulnerability to complications including orchitis. However, changes in either MMR2 VC or VE resulted in small absolute changes in expected number of orchitis cases (only about 2 cases prevented). While changes in overall vaccination strategy should therefore not be aimed at reducing mumps orchitis cases, it is critical to maintain already high MMR2 VC on college campuses to prevent not only mumps cases, but mumps complications like orchitis. Where not already in place, strategies could include establishing an immunization registry to track total student body VC, requiring documentation of vaccination or exemption, offering vaccines at every medical encounter and in non-traditional venues (e.g., student centers, dormitories), allowing students to be vaccinated without an appointment (Caleb et al., 2020), and using materials that emphasize the importance of vaccination to prevent not only mumps, but also orchitis, as incentive for vaccination.

This analysis is subject to several limitations. First, the number of mumps outbreaks with adequate data available for model parametrization was limited, favoring larger outbreaks more likely to include cases among under-vaccinated persons. However, our inferred distribution of mumps R_0_ is similar to the estimated range 3.6–4.5 from data from the pre-vaccine era (Edmunds et al., 2000). Second, these results are not generalizable to mumps outbreaks occurring outside of college campuses, particularly in communities with low VC. However, unlike outbreaks of other vaccine-preventable diseases, most recent U.S. mumps outbreaks have occurred on college campuses and in large part have occurred in highly vaccinated populations (Clemmons et al., 2019). Third, MMR2 VE estimates were obtained using the screening method, possibly resulting in higher or lower estimates from those obtained from case-control studies, depending on whether population VC was over- or underestimated. Fourth, due to data limitations, we estimated VE of ≥ 2 doses of MMR relative to under-vaccination (0–1 doses), which would be expected to underestimate MMR2 VE relative to no vaccination. These estimates are biased by the proportion of under-vaccinated students who received MMR1 and the proportion of fully vaccinated students who received MMR3 prior to the outbreak, though the latter likely represents a very small number. Because we used the screening method to estimate VE, the transmission model was also unable to account for MMR vaccination campaigns after outbreak onset, which may increase population VC. Finally, we made several simplifying assumptions to make the compartmental model tractable. We assumed population homogeneity in contact rates and risk of infection; we did not account for student subpopulations (e.g., fraternities/sororities, sports teams) known to have higher contact rates, increasing the potential to transmit mumps. We also did not account for incomplete case ascertainment, asymptomatic transmission of mumps virus, multiple introductions of mumps virus to the campus, or differential transmissibility by vaccination status of the case-patient. Lastly, we assumed mumps transmission stopped after outbreak day 62. In large outbreaks students may depart campus for the holidays during the incubation period and return while infectious, or mumps transmission may continue even with a lower on-campus population.

Despite the > 99% reduction in mumps cases by the early 2000 s in the United States, there has been an increase in mumps cases and outbreaks over the past 15 years, particularly among highly vaccinated populations in congregate settings like college campuses. Our study provides an estimated VE of 82.4–85.1% that novel vaccines or vaccination strategies must achieve to prevent large mumps outbreaks from occurring on college campuses, and further suggests that MMR2 VE among young adults may be lower than currently estimated. Knowing the VE threshold needed to prevent outbreaks will help to inform vaccine researchers and policy makers in developing future strategies.

## Supplementary Material

Supplementary material

## Figures and Tables

**Table 1:** Characteristics of 74 mumps college outbreaks reported to the Centers for Disease Control and Prevention during January 2016-June 2017, by inclusion in transmission model parameterization.

	Outbreaks included in model parameterization (N = 4)	Outbreaks with insufficient data to be included in model parameterization (N = 70)	P-value
	Median (range)	Median (interquartile range)	

Number of mumps cases reported	197 (28–1240)^a^	13 (6–26)	< 0.01^b^
Outbreak duration in days^c^	283 (110–751)^a^	71 (25–104)	< 0.01^b^
Percent population coverage with ≥ 2 MMR^d^ doses	97.5 (95.0–98.1)	98.6 (95.7–99.5)^e^	0.37^b^
Percentage of mumps case-patients with ≥ 2 MMR^d^ doses	92.4 (54.9–92.9)^a^	95.7 (88.8–100.0)^f^	0.16^b^
	**Number (%)**	**Number (%)**	
Use of MMR vaccination campaign	3 (75.0)^a^	27 (38.6)	0.18 ^g^

a For outbreaks for which control interventions included an MMR vaccination campaign, only pre-intervention data were used in model parameterization.

b Wilcoxon rank-sum test.

c Time from first mumps illness onset to last mumps illness onset.

d Measles, mumps, and rubella vaccine.

e Data available for 13 outbreaks.

f Data available for 68 outbreaks.

g Barnard’s exact test.

**Table 2:** Estimated two-dose measles, mumps, and rubella vaccine (MMR2) effectiveness against mumps in four mumps college outbreaks used in transmission model parameterization.

Outbreak	MMR2 vaccine effectiveness, % (95% confidence interval)

A	76.2 (63.7–84.4)
B	67.0 (47.2–80.4)
C	54.1 (42.2–63.5)
D	31.6 (0.0–67.1)^a^

a 95% confidence interval truncated at zero

**Table 3:** Proportion of 2000 outbreak simulations exceeding 20 and 100 mumps cases by theoretical vaccine effectiveness of a novel intervention against mumps, assuming constant vaccine coverage of 97.5% on a college campus with population 10,000.

Vaccine effectiveness against mumps, versus under-vaccination, %	Incremental vaccine effectiveness, versus MMR2^a^, %	Outbreaks with > 20 mumps cases, %	Outbreaks with > 100 mumps cases, %

60.5	0.0	40.6	18.3
73.1	31.9	19.2	5.0
78.2	44.8	10.9	2.0
82.4	55.4	5.0	0.7
85.9	64.3	2.0	0.3

a Two doses of measles, mumps, and rubella vaccine
