# Supplementary material for: Impact of vaccine effectiveness and coverage on preventing large mumps outbreaks on college campuses: Implications for vaccination strategy
Source: Epidemics. Author manuscript; Available in PMC 2025 Feb 21. (PMC11843670; doi:10.1016/j.epidem.2022.100594)
Supplement: Supplementary material [file NIHMS2057497-supplement-Supplementary_material.docx]

**Supplementary Material for *Impact of vaccine effectiveness and coverage on preventing large mumps outbreaks on college campuses: implications for vaccination strategy***

$$N_{V}=VC*N$$

$$N_{U}=N-N_{V}$$

$$N_{V}=S_{V}+E_{V}+I_{V}+R_{V}$$

$$\frac{dS_{V}}{dt}= -\gamma{*R}_{0}*\left( 1-VE \right)*S_{V}*\frac{I_{V}+I_{U}}{N}$$

$$\frac{dE_{V}}{dt}=\gamma*R_{0}*\left( 1-VE \right)*S_{V}*\frac{I_{V}+I_{U}}{N}-\tau*E_{V}$$

$$\frac{dI_{V}}{dt}=\tau*E_{V}-\gamma*I_{V}$$

$$\frac{{dR}_{V}}{dt}= \gamma*I_{V}$$

$$N_{U}=S_{U}+E_{U}+I_{U}+R_{U}$$

$$\frac{dS_{U}}{dt}= -\gamma{*R}_{0}*S_{U}*\frac{I_{V}+I_{U}}{N}$$

$$\frac{dE_{U}}{dt}=\gamma*R_{0}*S_{U}*\frac{I_{V}+I_{U}}{N}-\tau*E_{U}$$

$$\frac{dI_{U}}{dt}=\tau*E_{U}-\gamma*I_{U}$$

$$\frac{{dR}_{U}}{dt}= \gamma*I_{U}$$

**Figure S1.** Ordinary differential equations governing number of people in each compartment over time.

**Symbols and notes:**
N = total college population; held constant at 10,000 people across all simulations
VC = vaccine coverage (proportion of population fully vaccinated against mumps at the start of outbreak)
N_V_ = number of fully vaccinated people, total
N_U_ = number of under-vaccinated people, total
t = time since start of mumps outbreak
S_V_(t) = number of fully vaccinated people uninfected with mumps virus at time t; S_V_(0) = N_V_
E_V_(t) = number of fully vaccinated people infected with mumps virus, but not yet infectious, at time t; E_V_(0) = 0
I_V_(t) = number of fully vaccinated people infectious with mumps virus at time t; I_V_(0) = 0
R_V_(t) = number of fully vaccinated people recovered from mumps, no longer infectious, at time t; R_V_(0) = 0
S_U_(t) = number of under-vaccinated people uninfected with mumps virus at time t; S_U_(0) = N_U_ – 1
E_U_(t) = number of under-vaccinated people infected with mumps virus, but not yet infectious, at time t; E_U_(0) = 0
I_U_(t) = number of under-vaccinated people infectious with mumps virus at time t; I_U_(0) = 1
R_U_(t) = number of under-vaccinated people recovered from mumps, no longer infectious, at time t; R_U_(0) = 0
γ = recovery rate from infectiousness with mumps virus (inverse of duration of infectiousness)
R_0_ = mumps basic reproduction number
VE = vaccine effectiveness of full vaccination against mumps
τ = rate of progression from infection to infectiousness with mumps virus (inverse of time from infection to infectiousness)

**Table S1.** Distributions of epidemiological and clinical parameters used in compartmental model.

| **Parameter** | **Symbol** | **Distribution** | **References** |
| --- | --- | --- | --- |
| Total population | N | Constant: 10,000 people |  |
| Duration of infectiousness with mumps virus, in days | 1/γ | 1 + Gamma (shape=3.58, rate=0.628)  Median: 6.2 days  Interquartile range: 4.5–8.3 days | (1) |
| Time from infection to infectiousness with mumps virus, in days | 1/τ | Gamma (shape=96, rate=6.9)  Median: 13.4 days  Interquartile range: 12.5–14.3 days | (1-3) |
| Mumps basic reproduction number | R_0_ | Empiric: conditional on sampled γ and τ; fit to characteristics of sampled mumps outbreak (Table S1)  Median: 3.8  Interquartile range: 2.6–5.5 |  |
| Proportion of mumps cases among males |  | Constant: 0.5 |  |
| Proportion of vaccinated males with mumps who develop orchitis |  | Constant: 0.06 | (4) |
| Proportion of unvaccinated males with mumps who develop orchitis |  | Constant: 0.3 | (4) |

1. G. Henle, W. Henle, et al., Isolation of mumps virus from human beings with induced apparent or inapparent infections. *J Exp Med* **88**, 223-232 (1948).

2. F. A. Ennis, D. Jackson, Isolation of virus during the incubation period of mumps infection. *J Pediatr* **72**, 536-537 (1968).

3. T. C. Shope, J. C. Hierholzer, Isolation of mumps virus. *J Pediatr* **74**, 841-842 (1969).

4. M. A. Marlow *et al.*, "Mumps [chapter 9]" in CDC Manual for the Surveillance of Vaccine-Preventable Diseases. (Centers for Disease Control and Prevention, Atlanta, GA, 2021).


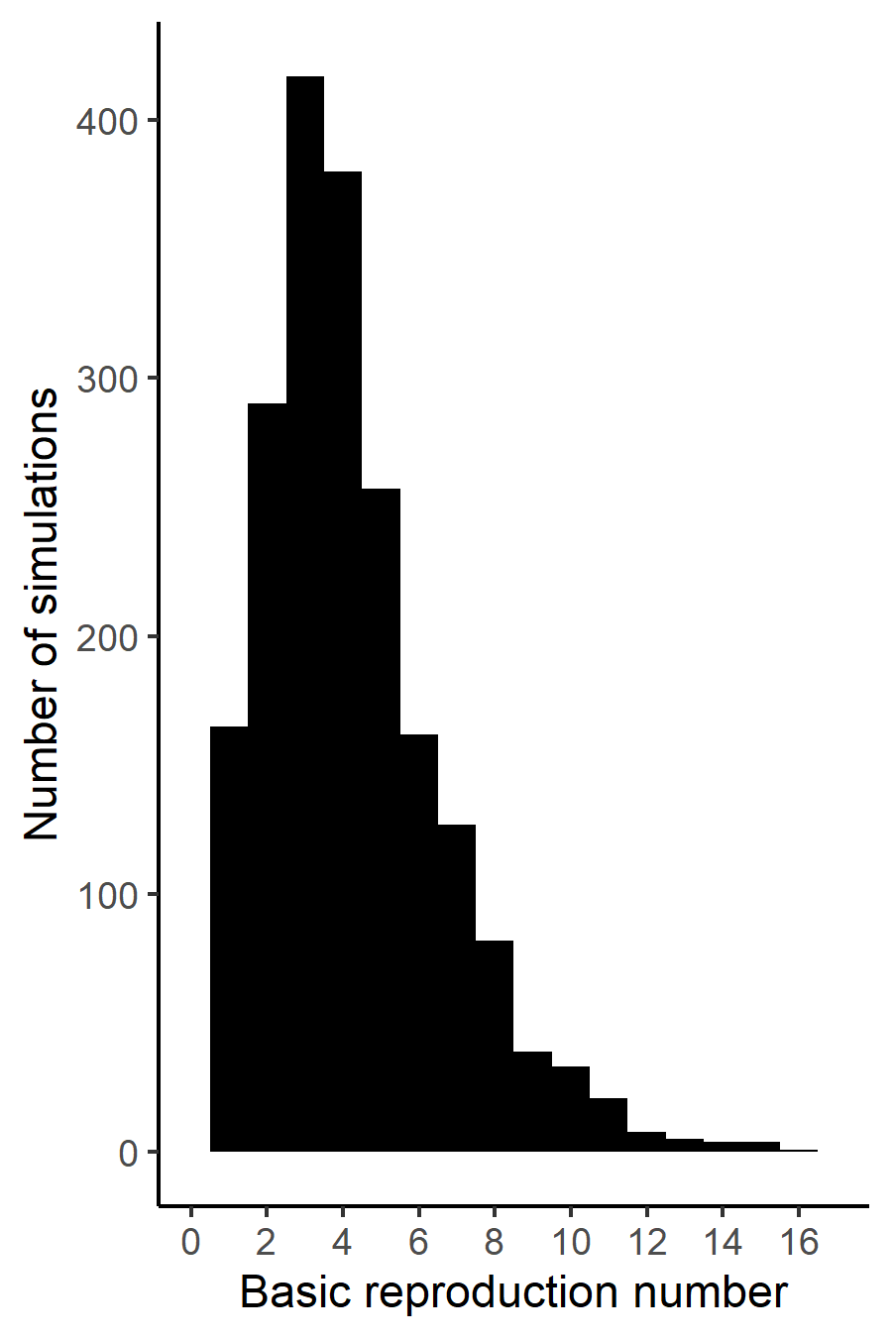


**Figure S2.** Frequency distribution of mumps basic reproduction number over 2,000 simulations of a compartmental model of mumps transmission on a college campus.

**Table S2.** Mean number of cases of mumps and mumps orchitis across 2,000 college outbreak simulations (population 10,000) by coverage and effectiveness of full vaccination with measles, mumps, and rubella vaccine.

| **VE (%)** | **Mean number of cases of mumps** | | | | **Mean number of cases of mumps orchitis** | | | |
| --- | --- | --- | --- | --- | --- | --- | --- | --- |
|  | **VC=90%** | **VC=96%** | ***VC=97.5%*** | **VC=99%** | **VC=90%** | **VC=96%** | ***VC=97.5%*** | **VC=99%** |
| 30 | 1482.8 | 1390.0 | *1367.1* | 1344.3 | 65.1 | 49.6 | *45.9* | 42.3 |
| 35 | 1244.4 | 1142.0 | *1116.9* | 1092.0 | 55.3 | 41.0 | *37.7* | 34.5 |
| 40 | 1018.1 | 909.4 | *882.9* | 856.7 | 46.0 | 33.0 | *30.0* | 27.1 |
| 45 | 807.3 | 696.3 | *669.6* | 643.4 | 37.2 | 25.6 | *23.0* | 20.5 |
| 50 | 615.9 | 507.8 | *482.3* | 457.6 | 29.1 | 18.9 | *16.7* | 14.7 |
| 51 | 580.3 | 473.5 | *448.5* | 424.2 | 27.5 | 17.7 | *15.6* | 13.6 |
| 52 | 545.7 | 440.4 | *415.9* | 392.2 | 26.0 | 16.6 | *14.5* | 12.6 |
| 53 | 512.2 | 408.7 | *384.7* | 361.6 | 24.6 | 15.4 | *13.5* | 11.7 |
| 54 | 479.7 | 378.2 | *354.9* | 332.4 | 23.2 | 14.3 | *12.5* | 10.7 |
| 55 | 448.3 | 349.1 | *326.5* | 304.8 | 21.8 | 13.3 | *11.5* | 9.9 |
| 56 | 418.1 | 321.4 | *299.5* | 278.6 | 20.5 | 12.3 | *10.6* | 9.0 |
| 57 | 389.0 | 295.0 | *274.0* | 253.9 | 19.2 | 11.4 | *9.7* | 8.3 |
| 58 | 361.1 | 270.1 | *249.9* | 230.7 | 18.0 | 10.5 | *8.9* | 7.5 |
| 59 | 334.4 | 246.6 | *227.3* | 209.0 | 16.8 | 9.6 | *8.2* | 6.8 |
| 60 | 308.9 | 224.5 | *206.1* | 188.7 | 15.6 | 8.8 | *7.4* | 6.2 |
| *60.5* | *296.6* | *213.9* | *196.0* | *179.2* | *15.1* | *8.4* | *7.1* | *5.9* |
| 61 | 284.7 | 203.8 | *186.3* | 169.9 | 14.5 | 8.0 | *6.8* | 5.6 |
| 62 | 261.7 | 184.4 | *167.9* | 152.5 | 13.5 | 7.3 | *6.1* | 5.0 |
| 63 | 240.0 | 166.4 | *150.8* | 136.4 | 12.5 | 6.6 | *5.5* | 4.5 |
| 64 | 219.5 | 149.7 | *135.1* | 121.5 | 11.5 | 6.0 | *5.0* | 4.1 |
| 65 | 200.3 | 134.2 | *120.5* | 107.9 | 10.6 | 5.4 | *4.5* | 3.6 |
| 66 | 182.2 | 119.9 | *107.1* | 95.4 | 9.8 | 4.9 | *4.0* | 3.2 |
| 67 | 165.3 | 106.8 | *94.9* | 84.0 | 9.0 | 4.4 | *3.6* | 2.9 |
| 68 | 149.6 | 94.7 | *83.6* | 73.6 | 8.2 | 4.0 | *3.2* | 2.5 |
| 69 | 135.0 | 83.6 | *73.4* | 64.2 | 7.5 | 3.5 | *2.8* | 2.2 |
| 70 | 121.4 | 73.5 | *64.2* | 55.7 | 6.8 | 3.2 | *2.5* | 2.0 |
| 75 | 67.8 | 35.9 | *30.1* | 25.0 | 4.1 | 1.7 | *1.3* | 1.0 |
| 80 | 34.2 | 15.2 | *12.2* | 9.7 | 2.4 | 0.8 | *0.6* | 0.5 |
| 85 | 15.4 | 6.1 | *4.9* | 4.0 | 1.3 | 0.4 | *0.3* | 0.3 |
| 88 | 9.3 | 3.8 | *3.1* | 2.6 | 0.9 | 0.3 | *0.3* | 0.2 |
| 90 | 6.7 | 2.9 | *2.4* | 2.1 | 0.7 | 0.3 | *0.2* | 0.2 |
| 95 | 3.2 | 1.7 | *1.5* | 1.4 | 0.4 | 0.2 | *0.2* | 0.2 |
| 99 | 2.1 | 1.3 | *1.2* | 1.1 | 0.3 | 0.2 | *0.2* | 0.2 |

**Abbreviations**
VE = vaccine effectiveness, VC = vaccine coverage

**Note:** Table values corresponding to median observed outbreak VC and VE are italicized.

**A**
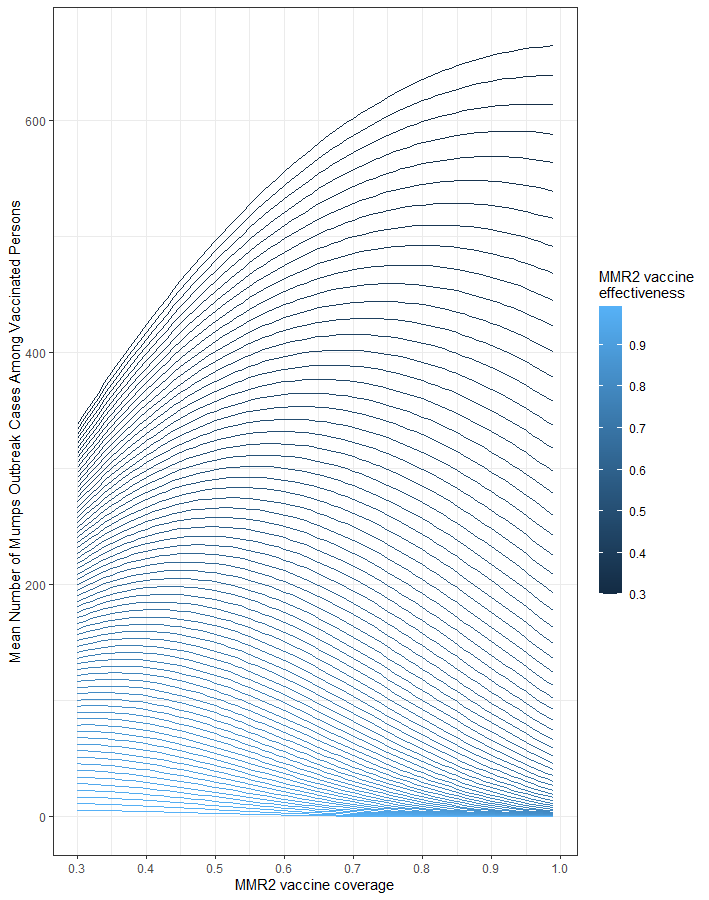
**B**
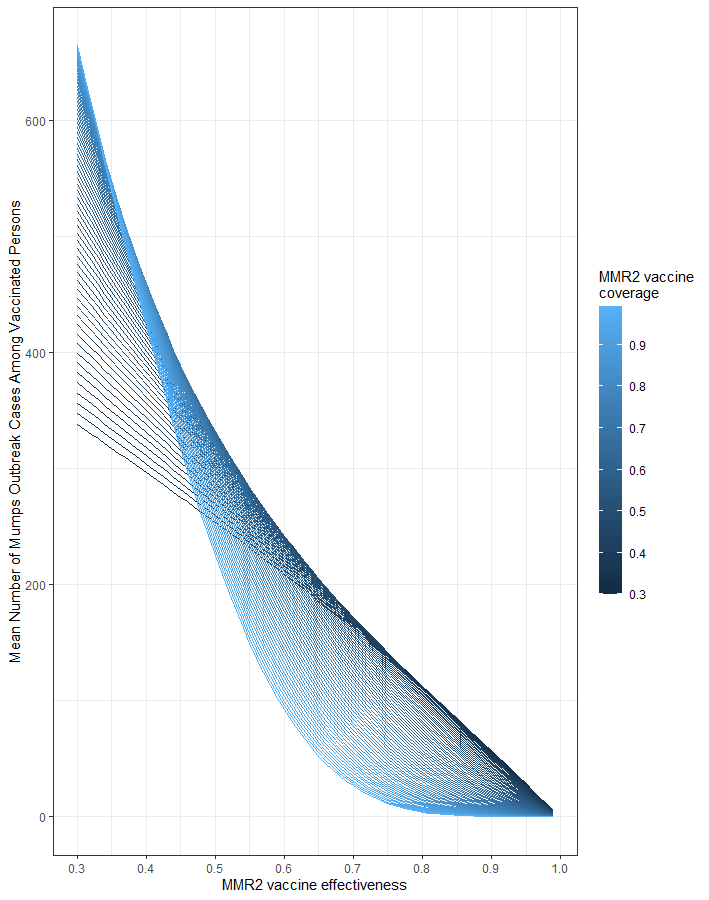


**Figure S3.** Impact of two-dose measles, mumps, rubella vaccine coverage and effectiveness against mumps on mean number of mumps cases among fully vaccinated persons across 2,000 outbreak simulations following introduction of an infected student to a college campus of population 10,000, (A) by vaccine coverage stratified by effectiveness and (B) by vaccine effectiveness stratified by coverage. MMR2 = two doses of measles, mumps, rubella vaccine

**A**
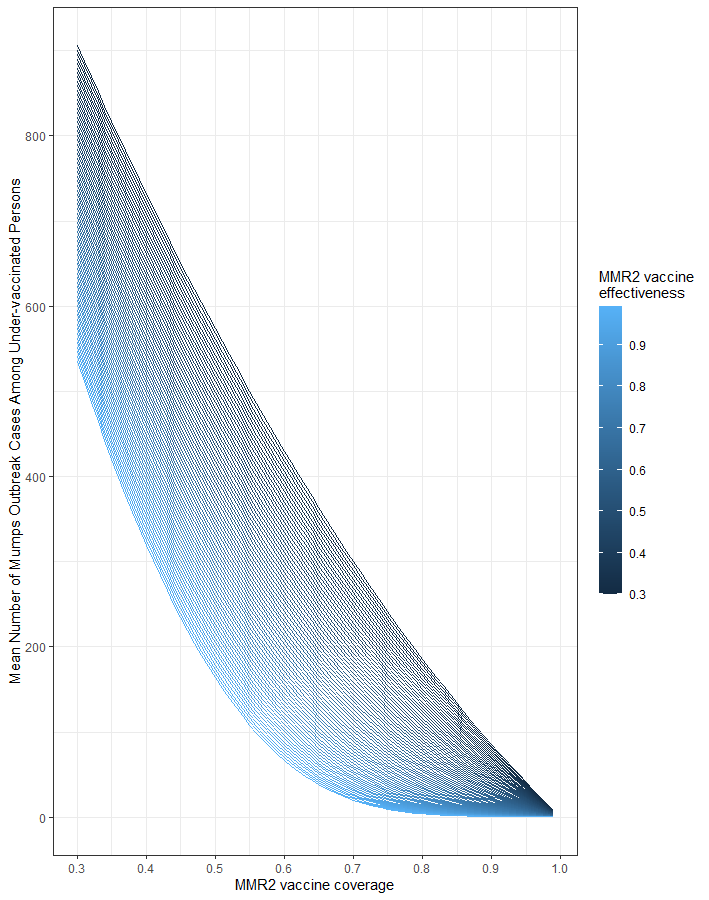
**B**
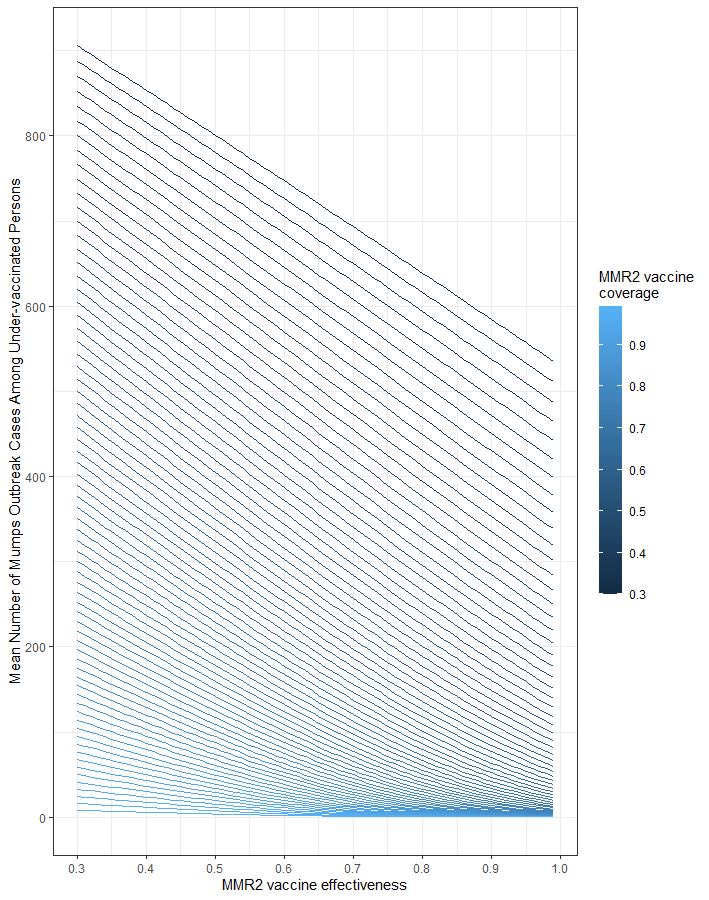


**Figure S4.** Impact of two-dose measles, mumps, rubella vaccine coverage and effectiveness against mumps on mean number of mumps cases among under-vaccinated persons across 2,000 outbreak simulations following introduction of an infected student to a college campus of population 10,000, (A) by vaccine coverage stratified by effectiveness and (B) by vaccine effectiveness stratified by coverage. MMR2 = two doses of measles, mumps, rubella vaccine

**A**
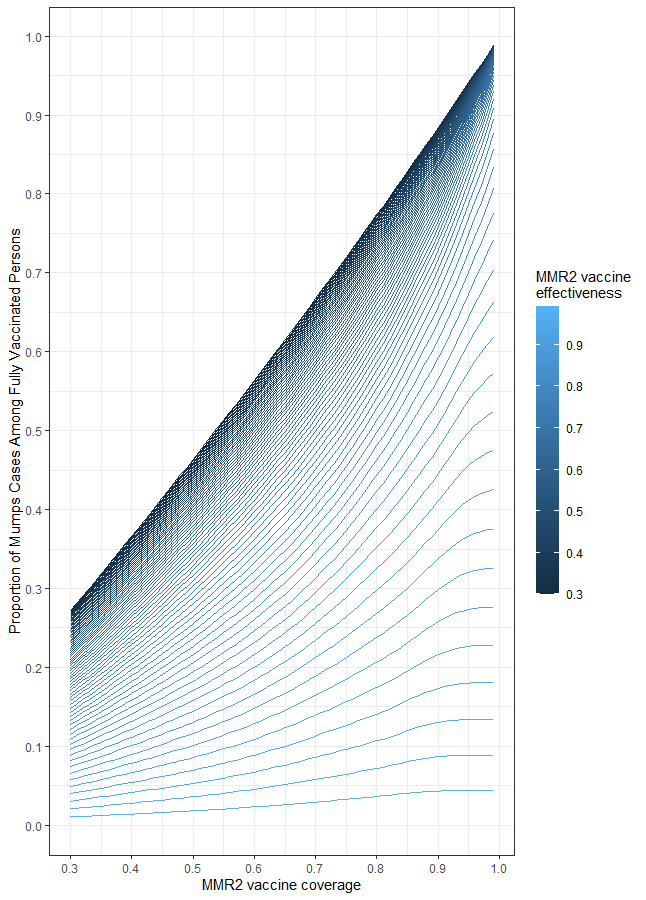
 **B**
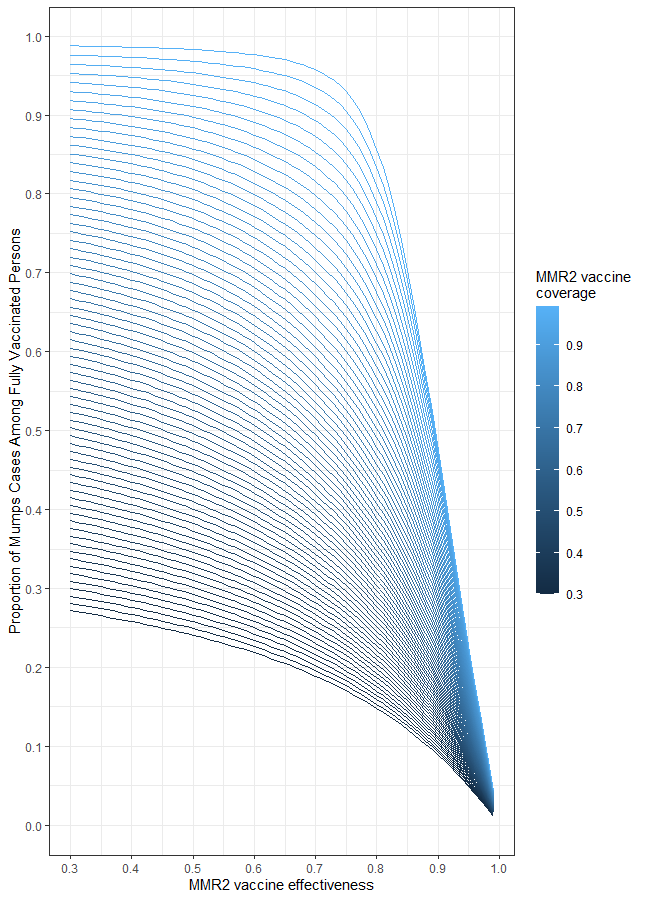


**Figure S5.** Impact of two-dose measles, mumps, rubella vaccine coverage and effectiveness against mumps on mean proportion of mumps cases among fully vaccinated persons across 2,000 outbreak simulations, (A) by vaccine coverage stratified by effectiveness and (B) by vaccine effectiveness stratified by coverage. MMR2 = two doses of measles, mumps, rubella vaccine
